# MIR143 Inhibits Steroidogenesis and Induces Apoptosis Repressed by H3K27me3 in Granulosa Cells

**DOI:** 10.3389/fcell.2020.565261

**Published:** 2020-10-19

**Authors:** Yuyi Zhong, Liying Li, Zitao Chen, Shuqi Diao, Yingting He, Zhe Zhang, Hao Zhang, Xiaolong Yuan, Jiaqi Li

**Affiliations:** ^1^Guangdong Provincial Key Lab of Agro-Animal Genomics and Molecular Breeding, National Engineering Research Centre for Breeding Swine Industry, College of Animal Science, South China Agricultural University, Guangzhou, China; ^2^Guangdong Provincial Key Laboratory of Laboratory Animals, Guangdong Laboratory Animals Monitoring Institute, Guangzhou, China

**Keywords:** ssc-miR-143-3p, H3K27me3, steroid hormones, cell apoptosis, porcine granulosa cells

## Abstract

The granulosa cell growth factor and apoptotic factor are two factors to determine follicular apoptosis. Whether ssc-miR-143-3p (MIR143) plays as an apoptosis factor in porcine granulosa cells (pGCs) remain unclear. This study tries to investigate what function of MIR143 is and how MIR143 gets these functions in pGCs from 3 to 5 mm medium-sized follicles. Firstly, 5′ RACE was used to identify the structure of MIR143, and *in situ* hybridization, qPCR, and DNA pull-down were employed to exhibit the spatio-temporal expression and transcriptional regulation of MIR143. Furthermore, ELISA, Western blotting, and flow cytometry were adopted to explore the functions of MIR143 in pGCs. It was found that MIR143 was an exonic miRNA located in host gene *LOC100514340* with an increasing expression during follicular growth. Moreover, MIR143 suppressed steroidogenesis related genes of HSD17β4, ER1, and PTGS2, negatively regulating estrogen, androgen, progesterone, and prostaglandin. MIR143 induced the apoptosis via activation of *BAX*-dependent *Caspase 3* signaling. Furthermore, H3K27me3 influenced the recruitment of transcription factors and binding proteins to repress MIR143 transcription. At last, H3K27me3 agonist with MIR143 inhibition activated steroidogenesis but repressed apoptosis. These findings suggest that H3K27me3-mediated MIR143 inhibition play a critical role in follicular atresia by regulating cell apoptosis and steroidogenesis, which will provide useful information for further investigations of H3K27me3-miediated MIR143 epigenetic regulation in follicular growth in mammals.

## Introduction

The follicular atresia is the fate of most follicles during folliculogenesis and development. During follicular development, follicular atresia occurs in the primordial, primary, secondary, and antral follicular stages in pigs (Evans, 2003). Generally, small follicles are more susceptible to atresia than large follicles (Lin and Rui, 2010). Extensive apoptosis of granulosa cells has been identified as the hallmark of follicular atresia (Guthrie et al., 1994; Guthrie and Cooper, 1996). It has been reported that the percentage of apoptotic granulosa cells and the ratio of G0/G1 in cell cycle were used to evaluate the proportion of heathy vs. atresia follicles (Guthrie et al., 1994; Guthrie and Cooper, 1996). Additionally, lower estradiol concentrations and higher progesterone concentrations in follicles are highly associated with follicular atresia, and the ratio of estradiol to progesterone (E2/PROG) is often used to identify follicular atresia (Guthrie and Cooper, 1996).

Multiple molecules participate in the process of porcine follicular atresia. Previous study has reported that during early atresia, CYP19 activity are significantly positively correlated with the amount of E2, while CYP11 and HSD3β promote atretic progression by increasing progesterone (Pan et al., 2012). A well-known mechanism of epigenetic regulation is post-transcriptional regulation of microRNA (miRNA), which plays critical role in the process of follicular atresia in pigs. For example, compared to healthy follicles, the expression of let-7 family members significantly declines in atretic follicles in pigs (Cao et al., 2015). Moreover, miR-26b promotes the apoptosis of granulosa cells by targeting the ataxia telangiectasia mutated gene (Lin et al., 2012), and miR-92a represses Smad7 to inhibit granulosa cell apoptosis and regulate follicular atresia in pigs (Liu et al., 2014).

The single strand of miRNA, ∼21 nt, is derived from an ∼70 nt pre-miRNA which is a precursor RNA splicing product of pri-miRNA transcribed from DNA. MiRNA genes are categorized according to their genomic location as intergenic (intergenic regions), intronic (intron regions), or exonic (exon regions) (Chen et al., 2012; Paczynska et al., 2015). The mechanism by which miRNA post-transcriptionally regulates a target is to bind with the seed-complementary sequences found in the 3′ UTR of target genes, resulting in translational repression or mRNA degradation (Cannell et al., 2008). It has been verified that an unstable 5′ terminal in the miRNA duplex is better for miRNA loading onto RNA-induced silencing complex (RISC) and recognizing the seed-complementary sequences in the 3′ UTR of the target to direct post-transcriptional repression (Hibio et al., 2012). The transcription process of miRNA genes also requires the regulation of transcription factors (TFs) (Xu et al., 2010), RNA polymerase (Lee et al., 2004; Borchert et al., 2006), and DNA methylation (Lodygin et al., 2008) as well as trimethylation of lysine 27 of histone 3 (H3K27me3). H3K27me3, the post-translational modification product that results from the action of polycomb repressive complex 2 (PRC2), enforces the transcriptional repression at the chromosome level (Pasini et al., 2008; Bender et al., 2013).

Aside from cell apoptotic stimulation in granulosa cells (Du et al., 2016), understanding about the gene structure, transcriptional regulation, and comprehensive biological functions of ssc-miR-143-3p (MIR143) is absent. The objective of this research is to investigate the genomic location, transcriptional regulation, and biological influence of MIR143, as this information will allow for further understanding of biological functions of MIR143 in porcine granulosa cells (pGCs).

## Materials and Methods

### Ethics Statement

Animal experiments were conducted according to the Regulations for the Administration of Affairs Concerning Experimental Animals (Ministry of Science and Technology, China). They were also approved by the Animal Care and Use Committee of the South China Agricultural University, Guangzhou, China (approval number 2018B116).

### Porcine Granulosa Cells Culture

Porcine ovaries were collected from a slaughterhouse in Guangzhou city. The ovaries were washed with cold PBS (Hyclone, United States) three times in slaughterhouse, and were then washed with pre-incubated PBS (37°C) containing 1% penicillin–streptomycin (Thermo Scientific, United States) twice under sterile conditions. The 3–5 mm medium-sized follicles were used to pGCs culture. The chosen follicles were with visible blood vessels, and full of translucence follicular fluid (Lin and Rui, 2010). The follicular fluid was absorbed with a 1 mL syringe and gently injected into a 15 mL sterile centrifugal tube containing 5 mL DMEM (Hyclone, United States). Total 8 mL follicular fluid-DMEM mixture were centrifuged to collect cell pellet at 1000 rpm for 5 min. Discarding supernatant, then the cell pellets were suspended and washed with DMEM twice. The cells were cultured in 15 mm^2^ cell culture flasks with 15 mL whole DMEM containing 10% FBS (Gibco, United States), 1% penicillin–streptomycin, and 89% DMEM. The culture medium was refreshed after 48 h. The oocytes were washed away because they were suspended in the medium. The pGCs were used in further research when they reached 90%–100% confluency at 24–48 h. The culture of pGCs was performed following the methods of reference (Yuan et al., 2019).

### The H3K27me3 Antagonist and Agonist, and MIR143 Mimics and Inhibitors

The GSK-126 (#1346574-57-9, MedChemExpress, United States) and GSK-J4 (#1373423-53-0, MedChemExpress, United States) were used as H3K27me3 antagonist and agonist, respectively. GSK-126 is a highly selective inhibitor of H3K27 methyltransferase EZH2 that reduces global H3K27me3 levels [46], whereas GSK-J4 is a potent dual inhibitor of H3K27me3/me2-demethylases JMJD3/KDM6B and UTX/KDM6A, which leads to an increase in the total nuclear H3K27me3 levels (Kruidenier et al., 2012). In this research, 6 nM GSK-126 was used to effectively inhibit H3K27me3, and 2 nM GSK-J4 was employed to effectively activate H3K27me3, as previously established (Zhong et al., 2020). The treatments for each group were triplicated, while for WB, each group contained two replicates.

The MIR143 mimics and inhibitors were designed and synthesized by RiboBio (Guangzhou, China). The concentrations of the MIR143 mimics (MIR143) were set at 25, 50, and 75 nM and the MIR143 inhibitors (siMIR143) were set at 50, 75, and 100 nM. The mimic controls (MNC) and inhibitor controls (siNC) had the same concentrations of mimic and inhibitor. The optimal transfected concentration was confirmed via stem–loop qPCR (Zhang et al., 2017) to quantify the MIR143 expression in pGCs. Both the transfection and qPCR of each group were technically triplicated. The primers of MIR143 are listed in Supplementary Table 1.

### MIR143 Gene Expression and Distribution

The expression profile of MIR143 was determined with stem–loop qPCR. Fourteen tissue types from the 180-day-old gilts (*n* = 3) included the heart, liver, spleen, lung, kidney, stomach, pancreas, large intestine, small intestine, cerebrum, cerebellum, hypothalamus, muscle, and ovaries. All tissues underwent RNA extraction following the Trizol extraction method. The RNA was quantified, and reverse transcription was performed for generation of cDNA using specific MIR143 RT primers (Supplementary Table 1). The expression of MIR143 (three replicates for each tissue) was measured by qPCR with primer information shown in Supplementary Table 1.

The operations of *in situ* hybridization (ISH) for MIR143 were performed according to the methods in Kloosterman et al. (2006). The antral follicles were classified as small-sized follicles (≤3 mm), mid-sized follicles (>3 and ≤ 5 mm) and large-sized follicles (>5 mm). There were three kind of follicles isolated from porcine ovaries (six ovaries from three female pigs): 8 mm antral follicles (AF), 5 mm antral follicles (BF), and 3 mm antral follicles (CF). The follicles were immersed in 4% paraformaldehyde for more than 2 h and used to prepare frozen sections. The probes of MIR143 (MI0013098, miRbase) were synthesized and labeled with horseradish peroxidase. The group with U6 probes was served as a positive control (PC), and the group with blank probes was a negative control (NC). Then, the probes of miRNA and U6 were hybridized in the follicular section for 16–20 h. Finally, the ISH results were visualized.

### GO and KEGG Pathways of MIR143 Candidate Targets

The sequences of hsa-miR-143-3p and MIR143 were aligned according to information from miRBase 22.11^1^. There were 46 verified targets of hsa-miR-143-3p in miRTarBase^2^, including *AKT1*, *AKT2*, *BCL2*, and *PTGS2* (Supplementary Table 2). The pathways of these 46 genes were analyzed using Cytoscape/ClueGO (3.7.2) (Adhikari et al., 2020). Furthermore, partial targets of hsa-miR-143-3p were considered to be MIR143 candidate targets, and possible combinations were predicted using RNAhybrid (version 2.2.1^3^) (Giegerich et al., 2004). The proteins AKT1 and KRAS were quantified by WB to evaluate the possibility of the post-transcriptional regulation of AKT1 and KRAS by MIR143. The antibodies of AKT1 (CST-4060s) and KRAS (CST-3965s) were purchased from Cell Signaling Technology (Boston, United States). The WB for AKT1 and KRAS was repeated twice.

### Hormone Measurement

Hormones in the pGC culture medium were measured via porcine Enzyme Linked Immunosorbent Assay (ELISA) Kits (Bai et al., 2017). The tested hormones included estrogen, androgen, progesterone, and prostaglandin. The Porcine Estrogen Kit (JL10508), Porcine Androgen Kit (JL26487), Porcine Progesterone Kit (JL36738), and Porcine Prostaglandin Kit (JL21995) were purchased from JiangLai Biotechnology (JiangLai, Shanghai, China). The hormone measurement included two parts. In part 1, the cell culture medium was collected 48 h after the transfection of MIR143, MNC, siMIR143, and siNC. In part 2, the cell culture medium was collected 48 h after the transfection of MIR143, MNC, siMIR143, and siNC, together with the treatment of antagonistic GSK-126 or agonistic GSK-J4. Each treatment in part 1 and part 2 featured three replications. All media were centrifuged at 3000 rpm for 20 min. A total of 500 μL of the supernatant was then collected from each sample for hormone detection while the cells were reserved for qPCR and WB analysis. The operations were performed following the Kit’s instructions. The hormone concentration of each sample was measured at 450 nm absorption using a microplate reader (iMark, BIORAD, Japan). The hormone analysis of each group featured four replications.

### Gene Expression Quantification

qPCR and Western blotting (WB) were used to reveal how MIR143 regulates hormone biosynthesis. The key molecules related to steroidogenesis include *FSHR* (NM 214386.3), *LHR* (JN 120794.1), *ER1* (NM 214220.1), *ER2* (NM 001001533.1), *CYP11A1* (NM 214427.1), *CYP19A1* (NM_214429), *HSD17*β*1* (EU 153250.1), *HSD17*β*4* (NM_214306.1), *PTGS2* (NM 214321.1), and *STAR* (NM 213755.2). The qPCR for each group comprised three replicates. All primers used in qPCR are listed in Supplementary Table 1.

The antibodies of PTGS2 (bs-0732R, BioSS ANTIBODIES, United States), CYP11A1 (bs-10099R, BioSS ANTIBODIES, United States), ER1 (bs-2098R, BioSS ANTIBODIES, United States), ER2 (bs-0116R, BioSS ANTIBODIES, United States), CYP19A1 (bs-1292R, BioSS ANTIBODIES, Woburn, United States), and HSD17β4 (bs-11296R, BioSS ANTIBODIES, Woburn, United States) were purchased from BioSS ANTIBODIES. The cells used for qPCR and WB were collected as previously indicated. The treatments included MIR143, MNC, siMIR143, and siNC transfection, in addition to human chorionic gonadotropin (hCG) stimulation, antagonistic GSK-126, and agonistic GSK-J4. The protein relative gray value = protein gray value/β-actin gray value in MIR143 and MNC with/without the hCG groups. The relative gray value = protein relative gray value of MIR143/protein relative gray value of MNC, together with antagonistic GSK-126 and agonistic GSK-J4.

### Detection of Cell Apoptosis

The cells used for the cell apoptotic flow cytometry (FMC) assay were grown in 6-well plates and were transfected with MIR143, MNC, siMIR143, and siNC, or transfected with MIR143, MNC, siMIR143, and siNC, along with antagonistic GSK-126 or agonistic GSK-J4. After 48 h, the cells were washed with room temperature PBS (Hyclone, United States) and digested using trypsin (Thermo Fisher Scientific, United States). Cells were centrifuged at 1000 rpm for 5 min and washed with PBS 3 times. Cells were suspended in a 500 μL binding buffer. Then, 5 μL of annexin V and 10 μL of PI (BioVision, United States) were added followed by thorough mixing, and incubation in the dark for 5 to 15 min. Cells were analyzed via FMC (Becton Dickinson, Co., San Jose, CA, United States) for 1 h. The apoptotic rate was calculated. The expressions of the cell apoptotic markers *BCL2* (XM_021099593.1), *BAX* (XM_003127290.5) and *Caspase 3* (NM_214131.1) (Zhu et al., 2015), and the proliferation markers *PNCA* (NM_001291925.1) were determine with qPCR. Information for all used primers is listed in Supplementary Table 1. The relative gene expression was calculated using the 2^–ΔΔct^ method. The FCM assay and qPCR of each group comprised three replicates.

### 5′ RACE of MIR143

5′ RACE technique was performed using the 5′-Full RACE kit D315 (Takara, Japan) and according to Song et al. (2010). Total RNA was extracted using the Trizol extraction method. RNA was dephosphorylated with alkaline phosphatase (16 U/μL, Promega, United States). The 5′ cap was removed using tobacco acid pyrophosphatase (TAP), followed by attachment of the 5′ RACE adaptor. The mRNA underwent reverse transcription with M-MLV reverse transcriptase (RNase H). The product of reverse transcription was used for outer PCR with the 5′ RACE outer primers, and the product was used for the inner PCR. The details of outer and inner primer are found in Supplementary Table 1. The final PCR product was purified and ligated into pMD19-T vector (Takara, Japan). The expected positive clones were extracted as recombinant plasmids. The plasmids were digested with *EcoR*I (Fermentas, Canada) and *Hind*III (Fermentas, Canada) to identify positive clones. The positive clones were sequenced by BGI (Shenzhen, China). More than three independent positive clones were used for sequence verification.

### Chromatin Immunoprecipitation PCR

H3K27me3 chromatin immunoprecipitation (ChIP) was carried out using the ChIP kit (#26156, Thermo Fisher, United States) and according to Shen et al. (2014). The pGCs were crosslinked by 1% paraformaldehyde and resuspended in PBS containing Halt cocktail. The supernatant was reserved following centrifuging after cell lysis. Then, 5 μL of lysed samples was used as input for analysis and 45 μL was used for further IP trials. Further, 10 μL aliquot of H3K27me3 antibody (#07-449, Millipore, Germany), 10 μL of the positive RNA polymerase II antibody, and 10 μL of negative IgG were added to each IP followed by incubation of the solutions overnight. Protein A agarose beads (10 μL) were washed with lysis buffer for three times and slowly mixed with the IP solution using a rotating mixer for 2 h. The mixture was washed with eluants and centrifuged at 4000 rpm for 5 min at 4°C. The ChIP eluant buffer was added to collect the binding DNA. The DNA was purified with a silica column and dissolved in an elution buffer. The DNA of the input, IgG, and H3K27me3 were used to amplify the H3K27me3 enriched region of MIR143. The RNA polymerase II IP and IgG IP were used to amplify the GAPDH enriched region. The PCR primers are listed in Supplementary Table 1. The qPCR of each group featured three replicates.

### MIR143 DNA Pull-Down

The previous results of the H3K27me3 ChIP-Seq in our lab showed that the promoter of MIR143 (± 2000 bp of TSS) was differentiated modified by H3K27me3 in 8 mm antral follicles vs. 5 mm antral follicles during porcine puberty (unpublished data). The procedures of ChIP-Seq followed the reference’s introductions (Sharma et al., 2017). The 1400 bp H3K27me3 enriched region of MIR143 was located in chromosome 2 at 150,578,101–150,579,500 bp (Sscrofa11.1). Therefore, DNA pull-down was performed to investigate protein enrichment in pGCs according to downregulate H3K27me3 with antagonistic GSK-126 and upregulate H3K27me3 with the agonistic GSK-J4. The blank group without any treatment was as a negative control. The DNA pull-down experiment was performed according to the methods from Jutras et al. (2012) and were performed as follows: (1) The nucleus proteins were extracted after treatments of 6 nM antagonistic GSK-126 and 2 nM agonistic GSK-J4 in whole DMEM medium for 48 h. The procedures of nucleus protein extraction were following the introduction of NE-PER^TM^ Nuclear and Cytoplasmic Extraction Reagent (#78835, Thermo Fisher, Waltham, MA, United States). (2) The streptavidin MIR143 DNA probes were purified and recycled after labeling with the streptavidin PCR primers (Gene Pharma, Shanghai, China). The primer sequences were shown in Supplementary Table 1. (3) The streptavidin MIR143 DNA probes were bundled with the magnetic beads to form magnetic bead-MIR143 DNA compounds. (4) Proteins from each group were added to the bead-MIR143 DNA compounds, forming magnetic bead-MIR143 DNA–protein compounds, which were analyzed by SDS-PAGE. (5) Proteins of each group were identified using the Q Exactive mass spectrometer (Thermo Scientific, United States). (6) The data for each group were cleaned by removing the background noise data from the magnetic bead–protein compounds. Clean proteins of each group = total proteins of each group - total proteins of the magnetic bead-protein compounds group. (7) BioVenn Software^4^ was to divide the specific- and common- enriched proteins of each group. Cytoscape (3.7.2.) was used to analyze the KEGG pathway enrichment (Adhikari et al., 2020). The TFs in each group were found by a comparison with the list of Homo sapiens TFs and predicted TFs of the H3K27me3 enriched region of MIR143 in the AnimalTFDB database^5^.

### Statistical Analysis

The data were expressed as the mean ± SEM. Statistical comparisons between the different groups were performed using a one-way ANOVA. Paired data were evaluated by Student’s *t*-test with the GraphPad Prism 7.0 software (San Diego, CA, United States). For this, ^∗^ indicates *P* < 0.05 and ^∗∗^ indicates *P* < 0.01. The correlation between MIR143 and host gene was analyzed with Pearson test. The Pearson’s correlation coefficient was tested with the function of “cor.test” in R “stats” package^6^.

## Results

### Genomic Location and Structure of the MIR143 Gene

**FIGURE 1:**
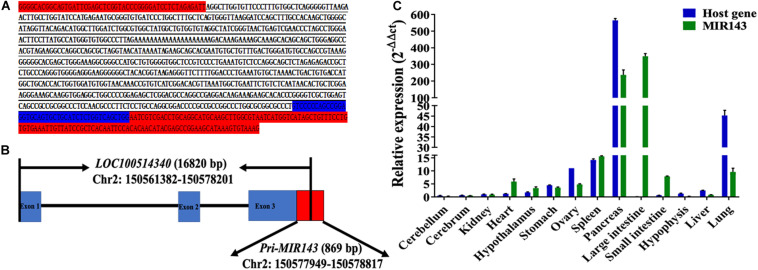
The structure, genomic location, and tissue expression patterns for MIR143. **(A)** The red-labeled text denotes sequences of pMD19-T. The blue-labeled text denotes partial sequences of pre-MIR143. The underlined text denotes sequences of 5′ RACE PCR amplification. **(B)** The structure of the *MIR143* gene in chromosome 2. Partial sequences of primary MIR143 (red block) were in exon 3 (blue block) of the *LOC100514340* gene. **(C)** The relative expression of MIR143 and host gene *LOC10051434* in 14 porcine tissues.

The 5′ RACE amplified sequences of MIR143 was 785 bp in length (Figure 1A) and located at 150,577,949–150,578,729 bp of chromosome 2. The alignment result between amplified sequences and the *Sscrofa11.1* genome was shown in Supplementary Figure 1. The transcription start site of the primary MIR143 (pri-MIR143) was nucleotide A at 150,577,949 bp of chromosome 2, and the entire pri-MIR143 sequences were located at 150,577,949–150,578,817 bp. In addition, MIR143 was found to locate in the exon 3 of the host gene *LOC100514340* (NC_010444.4, chromosome 2: 150,251,382–150,578,201 bp) (Figure 1B). The qPCR confirmed that the expression patterns of MIR143 were positively correlated with that of *LOC100514340* in 14 tissues (Pearson correlation, *P* < 0.05) (Figure 1C).

### The Expression Pattern of MIR143 in Antral Follicles

**FIGURE 2:**
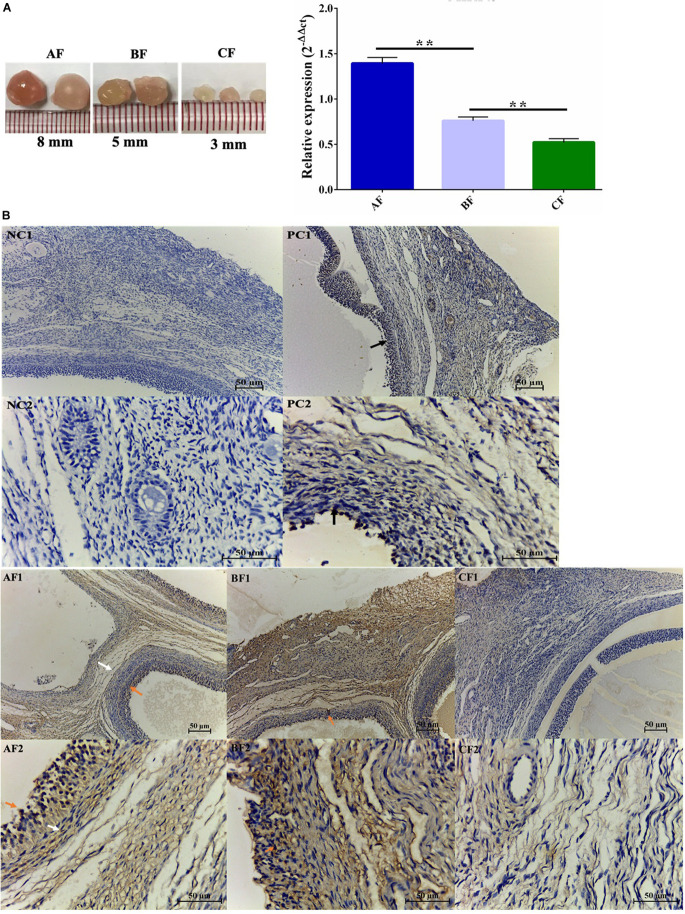
The temporal and spatial expression profile of MIR143 in porcine antral follicles. **(A)** qPCR results indicating the transcript levels of MIR143 in antral follicles throughout development. AF, BF, and CF indicated 8 mm antral follicles, 5 mm antral follicles, and 3 mm antral follicles. **P < 0.01. **(B)** Analysis of MIR143 expression by *in situ* hybridization. The black arrow in PC was the positive control U6. The red arrow and the white arrow denoted MIR143 in the follicular granulosa cells and theca cells, respectively. NC indicated the negative control with blank probes, PC was the positive control with U6 probes, and AF–DF are the experimental groups with MIR143 probes. AF1, BF1, CF1, and DF1 were images at 20 × amplification, and AF2, BF2, CF2, and DF2 were images at 40 × amplification.

The expression of MIR143 was increasing during antral follicle development (Figure 2A). The result was also corroborated by *in situ* hybridization (ISH), which also revealed that MIR143 higher expressed in both follicular granulosa cells (GCs) and theca cells of 5–8 mm follicles (AF: 8 mm follicles, BF: 5 mm follicles, Figure 2B), but lower expressed in 3 mm follicles (CF: 3 mm follicles, Figure 2B). Consequently, the increased expression of MIR143 might be related to its specific biological function during antral follicle development. As expected, the negative controls (NC) in slides NC1 and NC2 did not bind RNA probes, but the U6 positive control (PC) in slides PC1 and PC2 indicated that U6 bound to the U6 probes (Figure 2B).

### GO and KEGG Analysis of MIR143 Targets

**FIGURE 3:**
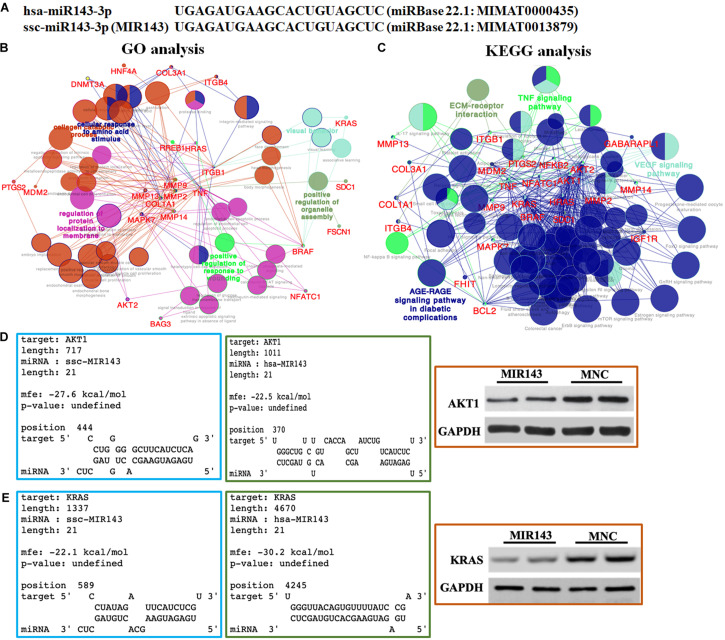
Bioinformatic prediction and functional analysis of MIR143 candidate targets. **(A)** Sequence alignment of human hsa-miR-143-3p and pig MIR143. **(B)** GO pathway analysis of hsa-miR-143-3p experimentally validated targets (MIR143 candidate genes). **(C)** KEGG pathway analysis of hsa-miR-143-3p experimentally validated targets (MIR143 candidate genes). **(D)** Predicted results from the RNAhybrid website (https://bibiserv.cebitec.uni-bielefeld.de/rnahybrid/) showed the possibility of hsa-miR-143-3p binding to human *AKT1* (verified, blue box) and pig MIR143 binding to pig *AKT1* (predicted, green box). Further, Western blotting (WB) showed the AKT protein expression when MIR143 was overexpressed in porcine GCs (red box, MNC indicated MIR143 mimic negative control). **(E)** Predicted results from the RNAhybrid website (https://bibiserv.cebitec.uni-bielefeld.de/rnahybrid/) showed the possibility of hsa-MIR143 binding to human *KRAS* (verified, blue box), and MIR143 binding to pig *KRAS* (predicted, green box). Further, WB showed the effect on KRAS protein expression when MIR143 was overexpressed in porcine GCs (red box, MNC indicated MIR143 mimic negative control).

According to the alignment results, the 21 nt mature MIR143 and human hsa-miR-143-3p were identical (Figure 3A). The 46 verified targets of hsa-miR-143-3p in miRTarBase, as candidate targets of MIR143, included *AKT1*, *MAPK7*, *KRAS*, *AKT2*, *MMP2*, and *PTGS2*. The GO pathway analysis showed that these targets engaged in the cellular response to the amino acid stimulus pathway (GO:0071230), the positive regulation of the organelle assembly pathway (GO:1902117), the regulation of protein localization to the membrane pathway (GO:1904375), and the positive regulation of the response to wounding pathway (GO:1903036) (Figure 3B).

The KEGG pathway analysis showed that the 46 candidate genes were enriched in four major pathways, namely the EM-receptor interaction pathway (KEGG: 04512), TNF signaling pathway (KEGG: 04668), VEGF signaling pathway (KEGG:04370), and AGE-RAGE signaling pathway in diabetic complications (K3GG: 04933) (Figure 3C). It was worth noting that the candidate genes participated in multiple signaling pathways related to follicular development and maturation, including progesterone-mediated oocyte maturation (KEGG: 04914; *AKT1*, *AKT2*, *BRAF*, *IGF1R*, and *KRAS*), the GnRH signaling pathway (KEGG: 04912; *HRAS*, *KRAS*, *MAPK7*, *MMP14*, and *MMP2*), the estrogen signaling pathway (KEGG: 04915; *AKT1*, *AKT2*, *BCL2*, *HRAS*, *KRAS*, *MMP2*, and *MMP9*), the FOXO signaling pathway (KEGG: 04068; *AKT1*, *AKT2*, *BRAF*, *GABARAPL1*, *HRAS*, *IGF1R*, *KRAS*, and *MDM2*), the mTOR signaling pathway (KEGG: 04150; *AKT1*, *AKT2*, *BRAF*, *HRAS*, *IGF1R*, *KRAS*, and *TNF*), the ErbB signaling pathway (KEGG: 04012; *AKT1*, *AKT2*, *BRAF*, *HRAS*, and *KRAS*), the NF-kappa B signaling pathway (KEGG: 04064; *BCL2, NFKB2, PTGS2*, and *TNF*), the IL-17 signaling pathway (KEGG: 04657; *MAPK7*, *MMP13*, *MMP9*, *PTGS2*, and *TNF*), and the prolactin signaling pathway (KEGG: 04917; *AKT1*, *AKT2*, *HRAS*, and *KRAS*). The RNAhybrid analysis predicted the possibility of a combination of pig MIR143 and the 3′ UTR of candidate genes related to follicular function, such as *AKT1*, *AKT2*, *KRAS*, *MMP2*, and *TNF* (Supplementary Figure 2). Proteins of two candidate targets, AKT1 and KRAS were repressed when MIR143 was overexpressed (Figures 3D,E). The results indicated that MIR143 might interact with multiple targets to play important epigenetic regulatory roles during antral follicle development.

### The Effect of MIR143 on Steroid Hormone Synthesis in pGCs

**FIGURE 4:**
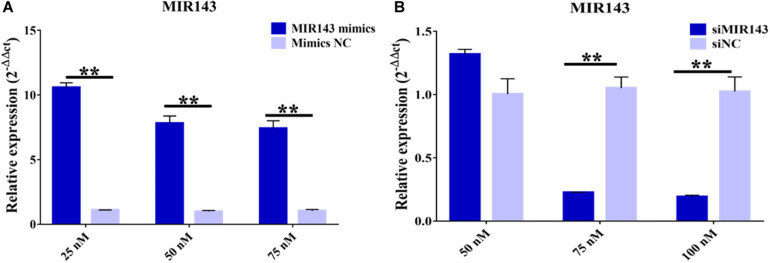
Effects of MIR143 mimics and inhibitors in porcine granulosa cells (pGCs). **(A)** MIR143 relative expression when transfected with 25, 50, and 75 nM MIR143 mimics or mimic controls. **(B)** MIR143 relative expression when transfected with 50, 75, and 100 nM inhibitor siMIR143 or inhibitor controls. **P < 0.01.

Treatments with 25, 50, and 75 nM of MIR143 mimics all significantly increased the levels of MIR143 in pGCs, with 25 nM MIR143 mimics stimulating the highest level of MIR143 expression. Considering the cell tolerance, 25 nM of MIR143 mimics was selected for further investigations (Figure 4A). In addition, for silencing assays, treatment with 75 nM of siMIR143 resulted in the highest inhibition efficiency than with 100 mM, and 75 nM of siMIR143, which was used for further analysis (Figure 4B).

**FIGURE 5:**
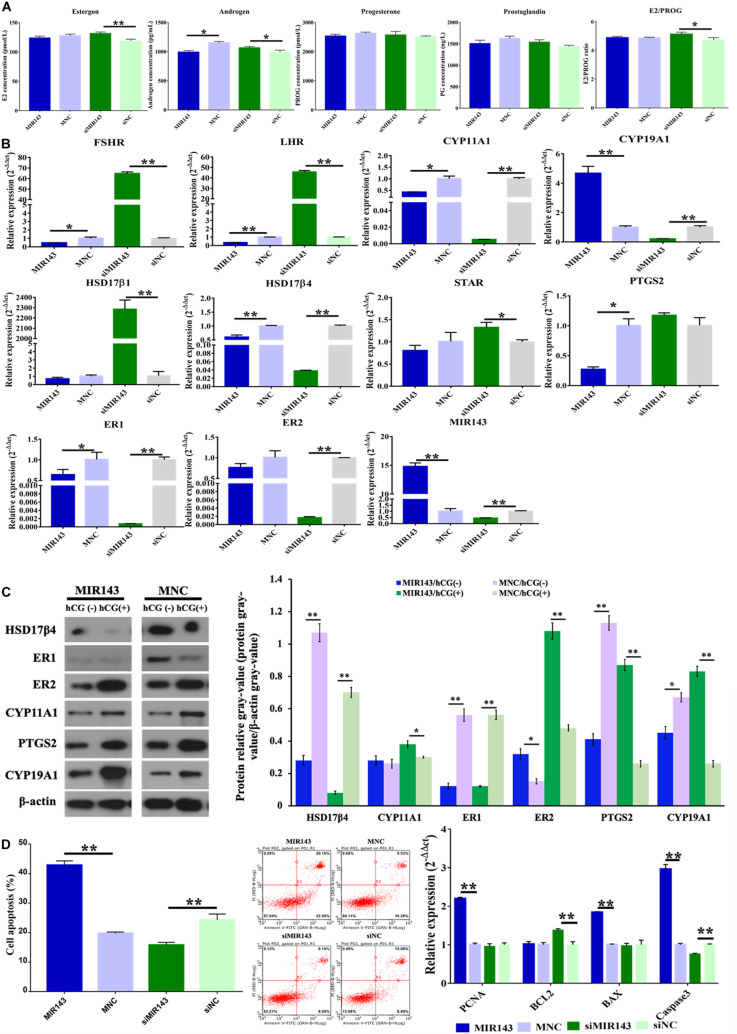
Effect of MIR143 on steroidogenesis and cell apoptosis in pGCs. **(A)** Hormone measurement in the pGC culture medium. MNC was the MIR143 mimic negative control, and siNC was the siMIR143 negative control. **(B)** Effect of MIR143 in the expression of key genes related to hormone biosynthesis. **(C)** Protein analysis of HSD17β4, ER1, ER2, CYP11A1, PTGS2, and CYP19A1 with or without hCG stimulation. The protein relative gray value = protein gray value/β-actin gray value. **(D)** Cell apoptosis analysis and mRNA levels of cell proliferation markers PCNA, and apoptosis biomarkers *BCL2*, *BAX*, and *Caspase 3*. *P < 0.05, **P < 0.01.

Compared to the mimic NC group, overexpression of MIR143 significantly decreased the concentration of androgen (*P* < 0.05) (Figure 5A). Compared to the inhibitor NC group, inhibition of MIR143 significantly increased the concentrations of estrogen (*P* < 0.01) and androgen (*P* < 0.05). The molecules related to steroidogenesis were identified with qPCR and WB under the treatments of MIR143 mimics and inhibitors. Compared to the mimic NC group, up-regulated MIR143 significantly decreased the mRNA levels of *FSHR* (*P* < 0.05), *LHR* (*P* < 0.01), *CYP11A1* (*P* < 0.01), *HSD17*β*4* (*P* < 0.01), *PTGS2* (*P* < 0.05), and *ER1* (*P* < 0.05), but markedly increased *CYP19A1* and *MIR143* (*P* < 0.01) (Figure 5B). Compared to siNC group, knocked-down MIR143 significantly increased the expression of *FSHR* (*P* < 0.01), *LHR* (*P* < 0.01), *HSD17*β*1* (*P* < 0.01), and *STAR* (*P* < 0.05), yet evidently repressed *CYP11A1*, *CYP19A1*, *HSD17*β*4*, *ER1*, *ER2*, and *MIR143* (*P* < 0.01) (Figure 5B). Additionally, increasing MIR143 inhibited the protein levels of HSD17β4, ER1, PTGS2, and CYP19A1, but enhanced the protein levels of ER2. Moreover, increased MIR143 together with hCG, resulted in the inhibition of HSD17β4 and ER1 at protein level, but activation of ER2, PTGS2, and CYP19A1 (Figure 5C). Consequently, the decreased of HSD17β4, ER1, and PTGS2 by up-regulating MIR143 might contribute to effects on steroidogenesis.

Furthermore, compared to the mimic NC group, MIR143 mimics significantly promoted cell apoptosis (Figure 5D). Compared to the inhibitor NC group, MIR143 inhibitors significantly inhibited cell apoptosis (Figure 5E). The expression of apoptotic biomarkers *BAX* (*P* < 0.01) and *Caspase 3* (*P* < 0.01), and cell proliferation biomarkers *PCNC* (*P* < 0.01) significantly increased when MIR143 was overexpressed. The mRNA expression of the cell apoptosis biomarker *Caspase 3* (*P* < 0.01) was significantly reduced, but *BCL2* evidently increased (*P* < 0.01), along with that the ratio of E2/PROG also increased upon treatment with siMIR143 (Figure 5A). These results indicated that MIR143 promoted cell apoptosis and inhibited the hormone synthesis in pGCs.

### H3K27me3 Repressed the Transcription of MIR143

**FIGURE 6:**
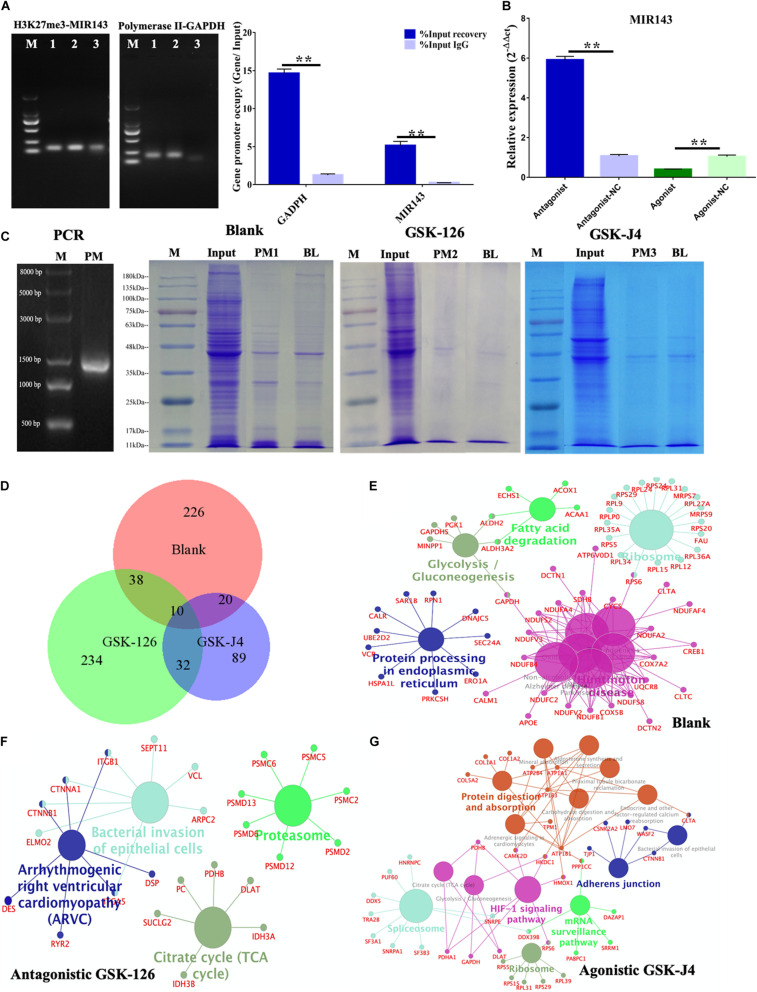
MIR143 DNA pull-down under treatments of antagonistic GSK-126 and agonistic GSK-J4. **(A)** ChIP-PCR results showing H3K27me3 occupancy in the H3K27me3 enriched region of MIR143. The 1, 2, and 3 represented PCR products of Input, IP, and IgG group of H3K27me3 and RNA polymerase II ChIP. **(B)** MIR143 relative expression with H3K27me3 and antagonistic GSK-126 or agonistic GSK-J4. The control groups for antagonistic-NC and agonistic-NC were 6 and 2 nM DMSO solvent, respectively. **(C)** The proteins in SDS-PAGE analysis of the blank group, antagonistic GSK-126 group, and agonistic GSK-J4 group in the DNA pull-down. **(D)** The common and specific proteins in the blank group, antagonistic GSK-126 group, and agonistic GSK-J4 group using BioVenn website (http://www.biovenn.nl). **(E–G)** KEGG pathways enriched in the blank, antagonistic GSK-126, and agonistic GSK-J4 groups. **P < 0.01.

The ChIP-PCR showed that H3K27me3 bound to the H3K27me3 enriched region of MIR143 (150,579,075–150,579,491 bp) (Figure 6A), and this observation indicated MIR143 might be targeted by H3K27me3 for transcriptional repression. In the positive control, the occupancy of RNA polymerase in the *GAPDH* promoter region was significantly higher than for the negative control of IgG. The quantification of H3K27me3 occupancy in the H3K27me3 enriched region of MIR143 presented the same trend as the positive control (Figure 6B). Furthermore, using 6 nM antagonistic GSK-126 significantly upregulated MIR143, and using 2 nM agonistic GSK-J4 significantly repressed MIR143. These results indicated that H3K27me3 occupied the enriched region of MIR143 to execute transcriptional repression, which resulted from GSK-126 and GSK-J4 impacting the yield of H3K27me3 and PCR2 activity.

The proteins binding to the 1400 bp H3K27me3 enriched region of MIR143 (150,578,101–150,579,500 bp of chromosome 2) were detected following a DNA pull-down assay (Figure 6C). A total 968, 983, and 715 raw proteins were output in the blank group, antagonistic GSK-126 group, and agonistic GSK-J4 group, respectively (Supplementary Table 3). After removing the background noise, 313, 340, and 158 proteins, respectively, were finally identified (Supplementary Table 3). Most identified proteins were specific to each group, with only a few were shared commonly among all three groups or between two groups (Figure 6D and Supplementary Table 3).

The KEGG analysis revealed five pathways that were enriched in the blank group, such as the fatty acid degradation pathway, the glycolysis gluconeogenesis pathway, and the ribosome pathway, including proteins of the RPL family such as GAPDH, APOE, CREB1, RPS6, and ACOX1 (Figure 6E). The pathways involved in the antagonistic GSK-126 group were the bacterial invasion of epithelial cell pathway and the arrhythmogenic right ventricular cardiomyopathy (ARVC) pathway, involving proteins of the PSMD family such as CTNNB1, CTNNA1, PHB1, and SUCLG2 (Figure 6F). Pathways gathered in the agonistic GSK-J4 group were the protein digestion and absorption pathway, the spliceosome pathway, the HIF-1 signaling pathway, the ribosome pathway, the mRNA surveillance pathway, and the adherent junction pathway, containing such RPS family proteins as CTNNB1, PUF60, and ATPA1 (Figure 6G). It was believed that some proteins executed important functions in the regulation of MIR143 transcription, especially the TFs.

Consequently, proteins of each group were compared to 1665 TFs in the list of *Homo* TFs. The results showed both increased and decreased H3K27me3 repressed TF recruitments in the H3K27me3 enriched region of MIR143 (Supplementary Table 4). Fifteen TFs were enriched in the blank group, namely CREB1, LRRFIP2, RUNX2, TFCP2, SSRP1, HMGB3, HMGA1, GATAD2A, FOXO6, CBFB, JUNB, CUX1, LYAR, and THYN1. Eight TFs were gathered in the antagonistic GSK-126 group, namely ST18, FOXN4, TFAM, LRRFIP2, GATAD2B, NFKB1, MTA2 and CBFB. Five TFs were acquired from the agonistic GSK-J4 group 5 TFs, namely TFAM, GATA1, CCDC88A, CASZ1, and CNBP. Furthermore, a total of 7523 TF binding sites of 508 TFs were predicted and were also considered as candidate TFs for the 1400 bp H3K27me3 enriched region of MIR143 (Supplementary Table 4). There were six (CREB1, RUNX2, CBFB, BRD3, TFCP2, and FOXO6), three (CBFB, NFKB1, and SMC1A), and three (GATA1, RB1, and DDX5) TFs match both for the predicted TFs and the proteins of each group. Consequently, the variants of endogenous H3K27me3 affect recruitment of TFs and binding proteins of the enriched region to regulate the transcription of MIR143.

### Function of the H3K27me3-MIR143 Signal in pGCs

**FIGURE 7:**
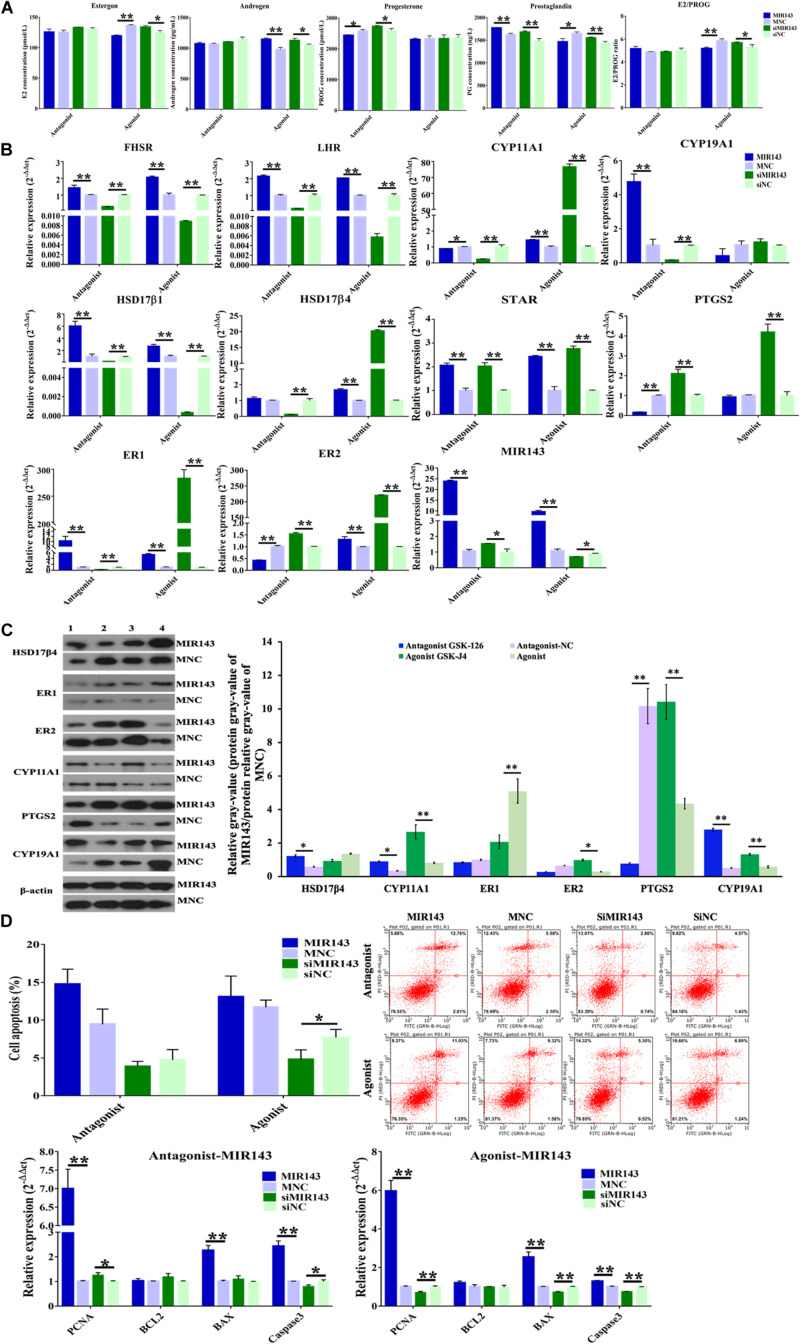
Function of H3K27me3-MIR143 signaling in pGCs. **(A)** Hormone measurement in pGC culture medium. Antagonist was H3K27me3 antagonist GSK-126; agonist was H3K27me3 agonist GSK-J4; MNC was MIR143 mimic negative control; siNC was siMIR143 negative control. **(B)** The effect of H3K27me3-mediated MIR143 signaling on the expression of steroidogenesis-related genes. **(C)** Protein analysis of CYP11A1, HSD17β4, ER1, ER2, and PTGS2 in the case of MIR143 overexpression together with the H3K27me3 antagonist or agonist. MNC is MIR143 mimic negative control. The numbers 1, 2, 3, and 4 represented the H3K27me3 antagonist GSK-126, antagonist NC, agonist GSK-J4, and agonist NC, respectively. The protein relative gray value = protein relative gray value of MIR143/protein relative gray value of MNC. **(D)** Cell apoptosis analysis and the expression of the proliferation markers *PNCA*, and the apoptotic markers *BCL2*, *BAX*, and *Caspase 3*. *P < 0.05, **P < 0.01.

Compared to the function of MIR143 mimics which inhibited steroid hormone (Figure 5A), MIR143 mimics together with antagonistic GSK-126 inhibited the concentration of progesterone (*P* < 0.05) but increased the concentration of prostaglandin (*P* < 0.01) (Figure 7A). The complementary recovery of agonistic GSK-J4 on MIR143 mimics suppressed the concentrations of estrogen (*P* < 0.01) and prostaglandin (*P* < 0.05) but increased the concentration of androgen (*P* < 0.01), all of which had significantly increased production by agonistic GSK-J4 with siMIR143 (Figure 7A).

The effects of H3K27me3-mediated MIR143 on the expression of steroidogenesis-related gene were elucidated and itemized as follows: (1) Compared to antagonistic GSK126/MNC, the accumulation of MIR143 via GSK-126 and MIR143 mimics significantly increased *FSHR*, *LHR*, *CYP19A1*, *HSD17*β*1*, *STAR*, *ER1*, and *MIR143*, but significantly decreased *CYP11A1*, *PTGS2*, and *ER2* (Figure 7B). At the protein level, HSD17β4, CYP11A1, and CYP19A1 were significantly increased, and PTGS2 was significantly decreased (Figure 7C). These results indicated that GSK-126 promoted MIR143 and activated the expression of steroidogenesis-related genes. (2) Compared to GSK126/siNC, complementary recovery with GSK-126 and siMIR143 markedly activated the mRNA levels of *STAR*, *ER2*, and *MIR143*, but markedly repressed *FSHR*, *LHR*, *CYP11A1*, *CYP19A1*, *HSD17*β*1*, *HSD17*β*4*, and *ER1* (Figure 7B). These results revealed that GSK-126 promoted MIR143 to repress the activation of siMIR143 for gene expression. (3) Although MIR143 was more highly expressed than GSK-J4/MNC, it was interesting that GSK-J4 together with MIR143 had a prominent activation on gene expression, including for *FSHR*, *LHR*, *CYP11A1*, *HSD17*β*1*, *HSD17*β*4, STAR*, *ER1*, and *ER2* (Figure 7B). At the protein level, ER1 was significantly decreased, and CYP11A1, ER2, PTGS2, and CYP19A1 were significantly increased (Figure 7C). (4) Compared to GSK-J4/siNC, the simultaneous inhibition of GSK-J4 and siMIR143 evidently increased *CYP11A1*, *HSD17*β*4*, *STAR*, *PTGS2*, *ER1*, and *ER2*, but significantly reduced *FSHR*, *LHR*, *HSD17*β, and *MIR143* (Figure 7B). Consequently, the effect of H3K27me3 in decreasing MIR143 resulted in the activation of steroidogenesis-related gene expression.

In both cases of antagonistic GSK-126 and agonistic GSK-J4, MIR143 induced cell apoptosis, while treatment with siMIR143 resulted in an anti-apoptotic effect (Figure 7D). In the case of GSK-126, the mRNA expressions of cell apoptosis markers showed that *BAX* (*P* < 0.01) and *Caspase 3* (*P* < 0.01) were significantly upregulated by MIR143 mimics, and the mRNA expression of *Caspase 3* (*P* < 0.05) was significantly reduced by MIR143 inhibitors. The cell proliferation marker *PCNA* (*P* < 0.01 and *P* < 0.05) was significantly promoted by both MIR143 mimics and MIR143 inhibitors. In the case of agonistic GSK-J4, the value of E2/PROG (*P* < 0.01) declined in response to MIR143 mimics and was promoted by siMIR143 inhibitors (*P* < 0.05) (Figure 7A). The cell apoptosis markers *BAX* (*P* < 0.01) and *Caspase 3* (*P* < 0.01) were significantly upregulated by MIR143 mimics and were significantly reduced by MIR143 inhibitors (*P* < 0.01). Cell proliferation marker *PCNA* (both *P* < 0.01) was enhanced by MIR143 mimics and was suppressed by MIR143 inhibitors (Figure 7D). The results highlight that cell apoptosis could be induced via increasing *BAX* and *Caspase 3* when MIR143 was overexpressed, and otherwise, promoted anti-apoptotic effects when MIR143 was knocked down in pGCs.

## Discussion

During the porcine follicular development (5 to 8 mm antral follicles), MIR143 was obviously detectable in both pGCs and theca cells and exhibited a trend of increasing expression going from small- to large-sized follicles (Figures 2A,B). Previous study has showed that bta-miR-143-3p, targeting *FSHR* in bovine granulosa cells, suppressed steroid hormone progesterone and E2 and also induced GC apoptosis (Zhang et al., 2017, 2019). Mmu-miR-143-3p, located in the primary, secondary, and antral follicles, negatively regulated FSH-induced estradiol production and cell apoptosis via targeting of KRAS (Zhang et al., 2017). In this research, higher MIR143 levels also inhibited *FSHR* and the steroid hormones progesterone and E2 in pGCs from medium-sized follicles (Figures 5A,B), which was consistent with previous research (Pasini et al., 2008). Hormones of estrogen, progesterone, and androgen were suppressed. We speculated that the decreased estrogen and androgen were caused by the decreased levels of precursor progesterone (LaVoie, 2017), and the decline in prostaglandin also limited progesterone synthesis in human granulosa cells (McNatty et al., 1975).

The non-coding miRNA of the RNA-induced silencing complex (RISC) complementarily bound to target 3′ UTR, mediating translation suppressing and even RNA degradation (Park and Shin, 2014). Considering that hsa-miR-143-3p was identical to MIR143, the potential biological functions of MIR143 were predicted according to the hsa-miR-143-3p verified targets in the KEGG pathway (Figure 3). It was found that many genes in multiple pathways, such as *AKT1*, *AKT2*, *MMP2*, and *KRAS*, were engaged in ovarian functions, including progesterone-mediated oocyte maturation (KEGG: 04914), the GnRH signaling pathway (KEGG: 04912), estrogen signaling pathway (KEGG: 04915), and the FOXO signaling pathway (KEGG: 04068). The results of RNAhybrid and WB showed that MIR143 might bind to 3′ UTR of *AKT* and *KRAS* to repress protein translation (Figures 3D,E), which provided minor evidence that *AKT* and *KRAS* might be MIR143 downstream targets. AKT presented in oocytes, granulosa cells, and theca cells in human follicles at each growing stage, and luteal cells and primordial follicles (Goto et al., 2007). The research verified that FSH regulated the B-cell lymphoma-2 (BCL2)-interacting modulator of cell death (Bim) via the PI3K/AKT/FOXO3 pathway and induced PGC apoptosis during follicular atresia (Wang et al., 2012). Members of the matrix metalloproteinases (MMPs), here MMP2 and MMP9, were important for follicular development, and were expressed throughout folliculogenesis and up to ovulation (Basini et al., 2011; Kim et al., 2014). Previous reports concerning the effect of KRAS in porcine follicular development were limited, but it was found that oncogenic KRAS regulated the granulosa cell cycle and the PI3K and MAPK pathways, mediating gonadotropin-induced events associated with ovulation in a mouse model (Fan et al., 2009). The results indicated that MIR143 might be an important epigenetic factor for inducing granulosa cell apoptosis to take part in follicular development, atresia, and maturation according to its multiple targets (Supplementary Figure 2). The increasing expression pattern of MIR143 during follicular was required for regulating target genes’ functions in certain biological processes.

This research has verified that MIR143 negatively regulates hormone synthesis and induces cell apoptosis in pGCs from medium-sized follicles. Overexpressed MIR143 inhibited estrogen, androgen, progesterone, and prostaglandin, though only androgen was observed significantly decreased in here (Figure 5A). One possible scenario explaining the molecular regulation of hormone synthesis by MIR143 was that the overexpression of MIR143 would downregulate the expression of a series of genes, including *FSHR*, *LHR*, *HSD17*β*1*, *STAR*, and *PTGS2* (Figure 5B), among which *HSD17*β*1*, *STAR*, and *PTGS2* are the key genes regulating the biosynthesis of steroid hormones (LaVoie, 2017). Consequently, the inhibition of hormone synthesis might be related to the repressed expression of MIR143. *FSHR* (Pasini et al., 2008) and *PTGS2* (Kim et al., 2011) have been proven to be the targets of MIR143 and affect the concentration of E2 and prostaglandin. MIR143 was observed to stimulate cell apoptosis through the significantly observed upregulation of *BAX* and *Caspase 3* (Figure 5D). MIR143 targeted *BCL2* to inhibit proliferation but promoted the apoptosis in HeLa cells (Liu et al., 2012) when MIR143 was overexpressed. BCL2 was not significantly downregulated in response to MIR143 overexpression but was significantly upregulated by MIR143 inhibition. This research inferred that MIR143 might activate *BAX*-dependent of *Caspase 3* signaling to stimulate cell apoptosis.

In this study, MIR143 has been characterized as an exonic miRNA, locating in exon 3 of the host gene *LOC100514340* on chromosome 2 (Figures 1A,B). It was reported that intronic and exonic miRNAs are both expressed in parallel with their host gene (Rodriguez et al., 2004). Here, we observed that MIR143 and host gene *LOC100514340* shared the correlated expression patterns in most of the assayed tissues (9 of 14), but they exhibited a special spatial expression pattern in some tissues (Figure 1C), which is assumed to coincide with the requirements of certain biological processes. It was reported that MIR143 is most abundant in smooth muscle and fibroblasts, but lower in lymphocytes, endothelial cells, epithelial cells, and red blood cells (Kent et al., 2014).

It has also demonstrated that H3K27me3 affects the recruitments of TFs and binding proteins to influence MIR143 transcription. Various factors regulate miRNA gene transcription, including the recruitment of RNA polymerase II (Kirmizis et al., 2004), RNA polymerase III (Borchert et al., 2006), TFs (Woods et al., 2007; Xu et al., 2010), epigenetic factors such as DNA methylation (Bandres et al., 2009) and histone post-transcription regulation (Agirre et al., 2009). It was verified by ChIP-PCR that H3K27me3 occupied in enriched region of MIR143, acting as a transcriptional repressor (Figures 6A,B). Moreover, the increasing expression pattern was negative correlation with H3K27me3 decreasing expression during follicular development (Zhong et al., 2020). The methylation of H3K27 into H3K27me3 is catalyzed by histone methyltransferase EZH2, which is a member of the polycomb repressive complex 2 (PRC2), also results in PRC2 binding to the chromatin of upstream genes and the transcription start site to switch off gene transcription. The functional PRC2 family includes members SUZ12, EED, and EZH2 (Kirmizis et al., 2004; Hansen et al., 2008). The transcriptional silencing mechanism under the colocalized recruitment of H3K27me3 and PRC2 to chromatin is related to polynucleosomic compaction (Margueron et al., 2008; Simon and Kingston, 2009) and DNA methylation (He et al., 2011). Interestingly, a previous study reported that androgen-induced mouse miR-101 targeting resulted in repression of EZH2, which led to a reduction in H3K27me3 occupancy of the promoter of the LH-induced TF RUNX1 functioning in follicular maturation and ovulation (Ma et al., 2017). In response to hCG stimulation, increased CYP11A1 led to reduced H3K27me3 occupancy in the CYP11A1 promoter region (Okada et al., 2016). According to the influence of H3K27me3 in follicular steroidogenesis and development, it is believed that the variation in the increase and decrease of endogenous H3K27me3 affect the recruitment of TFs and binding proteins to regulate MIR143 transcription and function in pGCs during follicular growth (Figures 6D–G).

Usage of GSK-126 and GSK-J4 globally repressed and increased nuclear H3K27me3 and PRC2 activity in pGCs, respectively, which highlighted the increasingly complicated situation of steroidogenesis-related gene regulation mediated by H3K27me3-mediated MIR143 signaling. As reported by previous report, GSK-126 inhibited the H3K27 methyltransferase EZH2, leading to a decrease in nuclear H3K27me3 (McCabe et al., 2012), while GSK-J4 inhibited H3K27me3/me2 demethylases to increase nuclear H3K27me3 (Kruidenier et al., 2012), and both led to variations in PRC2 activity and the subsequent transcription of its globally targeted genes. Using antagonistic GSK-126, for example, released the transcriptional repression of H3K27me3, though it did not intensify gene repression due to MIR143 overexpression (Figure 4B), but did upregulate the expression of numerous genes, including *FSR*, *LHR*, *HSD17*β*1*, and *ER2* (Figure 7B). The complementary recovery of antagonistic GSK126 with siMIR143 and agonist GSK-J4 led to different effects, with the former exhibiting a trend of inhibiting steroidogenesis-related gene expression, and the latter tending to stimulate gene expression (Figure 7B). These results indicated that H3K27me3-mediated-MIR143 signaling played an important role in steroidogenesis of pGCs.

The H3K27me3-mediated MIR143 signaling also exhibited effects on cell apoptosis with only small differences in cell apoptotic rate between agonistic GSK-126 and agonistic GSK-J4 treatments (Figure 7D). One possible explanation could be related to the expression of MIR143 and cell apoptotic biomarkers under the effects of antagonistic GSK-126 and agonistic GSK-J4. The results of cell apoptosis assays showed that MIR143 induced apoptosis in both cases where H3K27me3 was either reduced or increased through the upregulation of *BAX* and *Caspase 3* (Figure 7D). Neuronal cell death has been demonstrated to be a result of BAX-dependent Caspase 3 activation, inducing cell death (Cregan et al., 1999) due to BAX-induced polymerase (ADP-ribose) cleavage (Kagawa et al., 2001). Another possible explanation for the observed results could be that using agonistic GSK-126 and agonistic GSK-J4 shaped the chromatin of pGCs and affected the transcription of many genes (Okada et al., 2016; Ma et al., 2017) and not just MIR143, and the observe effects are due to alterations in the expression of these genes.

## Data Availability Statement

The raw data supporting the conclusions of this article will be made available by the authors, without undue reservation, to any qualified researcher.

## Ethics Statement

The animal study was reviewed and approved by The Animal Care and Use Committee of the South China Agricultural University, Guangzhou, China (approval number 2018B116).

## Author Contributions

YZ and JL: conceptualization. ZC and SD: data curation. YZ and LL: formal analysis and investigation. HZ, XY, and JL: funding acquisition. YZ and YH: methodology. ZZ, HZ, and XY: project administration. ZC and SD: software. JL: supervision. ZZ and XY: validation. YZ, LL, and YH: visualization. YZ: writing—original draft. YZ, ZZ, XY, and JL: writing—review and editing. All authors contributed to the article and approved the submitted version.

## Conflict of Interest

The authors declare that the research was conducted in the absence of any commercial or financial relationships that could be construed as a potential conflict of interest.

## Footnotes

1 http://www.mirbase.org/

2 http://mirtarbase.mbc.nctu.edu.tw/php/search.php

3 https://bibiserv.cebitec.uni-bielefeld.de/rnahybrid

4 http://www.biovenn.nl

5 http://bioinfo.life.hust.edu.cn/AnimalTFDB#!/

6 https://www.rdocumentation.org/packages/stats/
