# Supplementary material for: MIR143 Inhibits Steroidogenesis and Induces Apoptosis Repressed by H3K27me3 in Granulosa Cells
Source: Front Cell Dev Biol. 2020 Oct 19;8:565261. doi: 10.3389/fcell.2020.565261 (PMC7604341; doi:10.3389/fcell.2020.565261)
Supplement: Supplementary file 1 [file Table_1.DOCX]

Supplementary Material

## Supplementary Figures


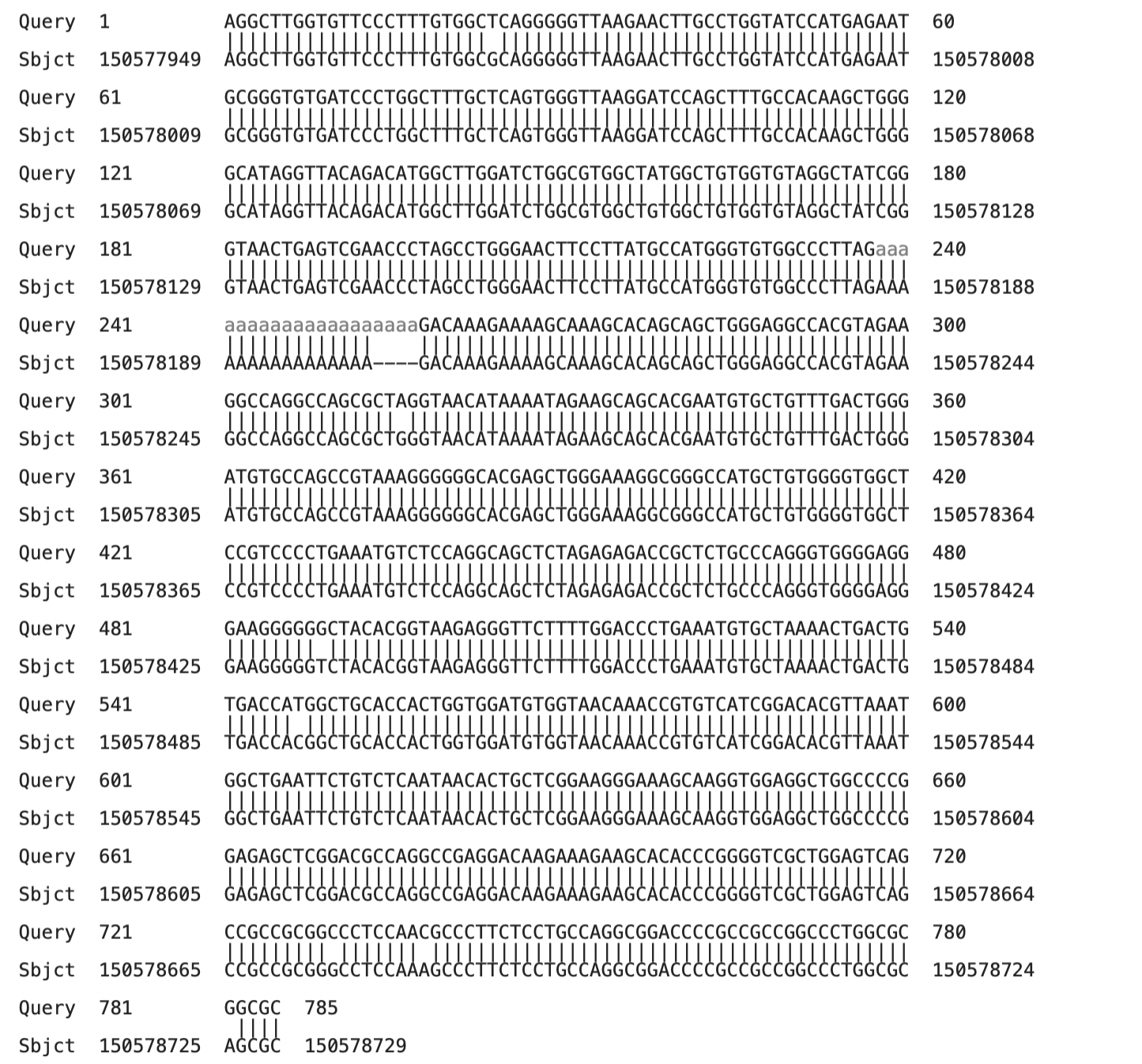


**Supplementary Figure 1.** The alignment of pig ssc-miR-143-3p (MIR143) 5'RACE and Sscrofa 11.1 reference genomic sequences. The result showed the MIR143 gene located in chromosome 2. And the primary transcription started site at 150577949 of nucleotide A. The Query were the 5'RACE sequences, and the Sbjct indicated Sscrofa 11.1 reference genomic sequences.

**A. AKT1**


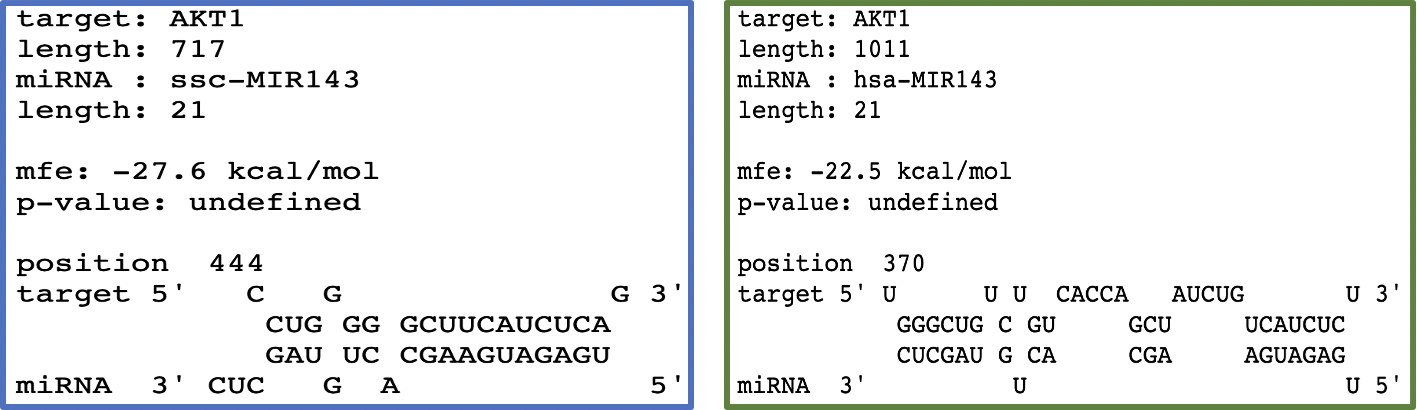


**B. AKT2**


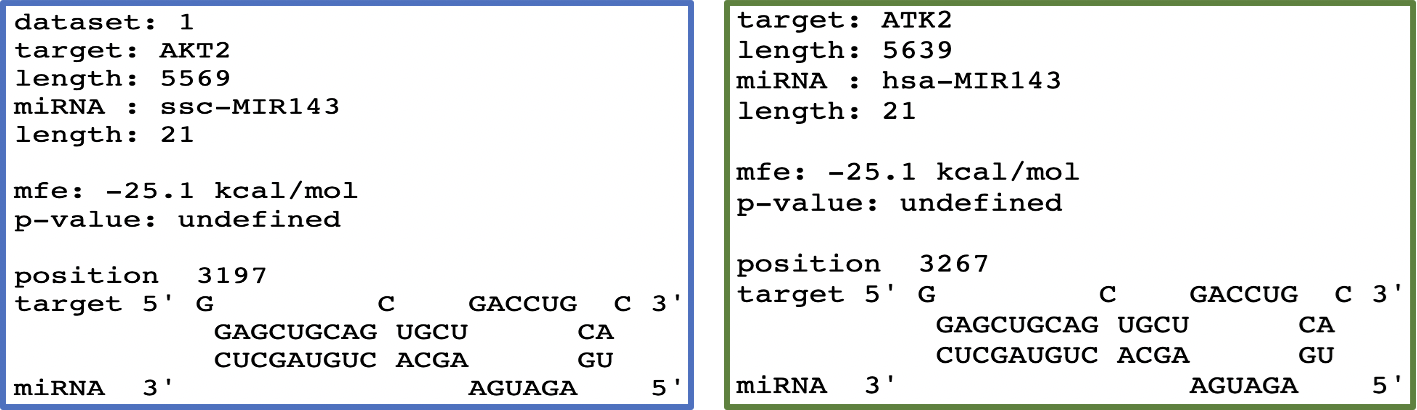


**C. BCL2**


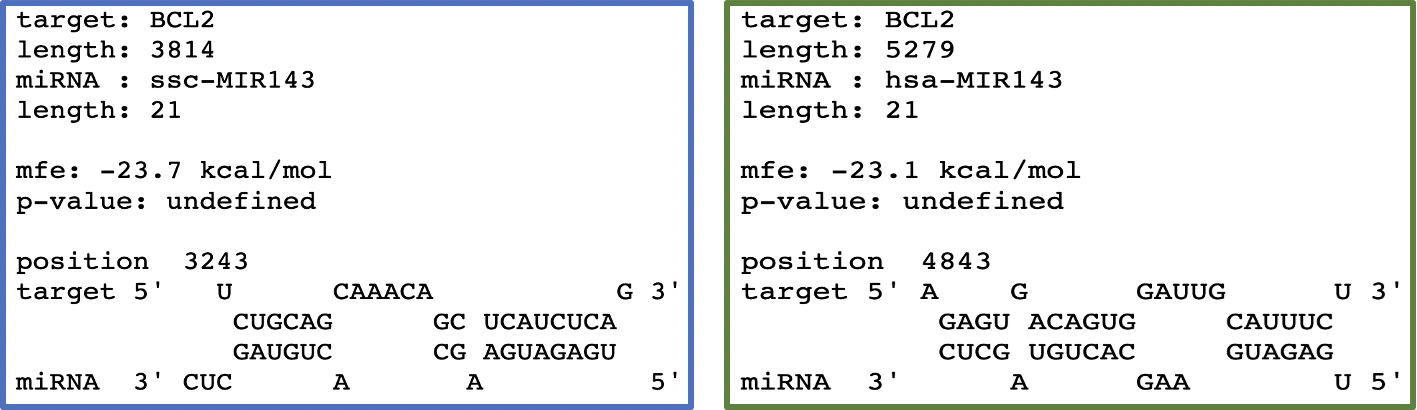


**D. BRAF**


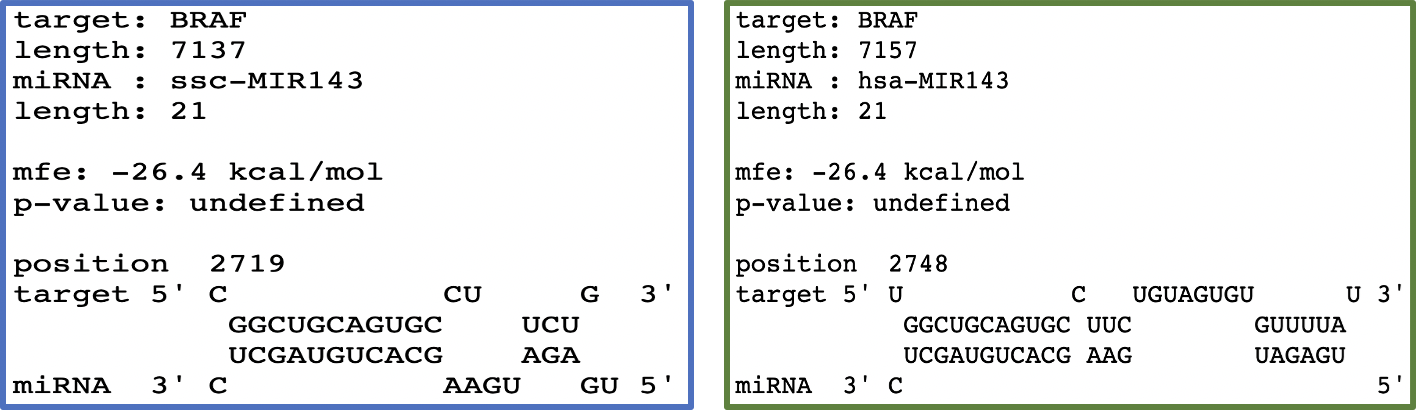


**E. GABARAPL1**

**
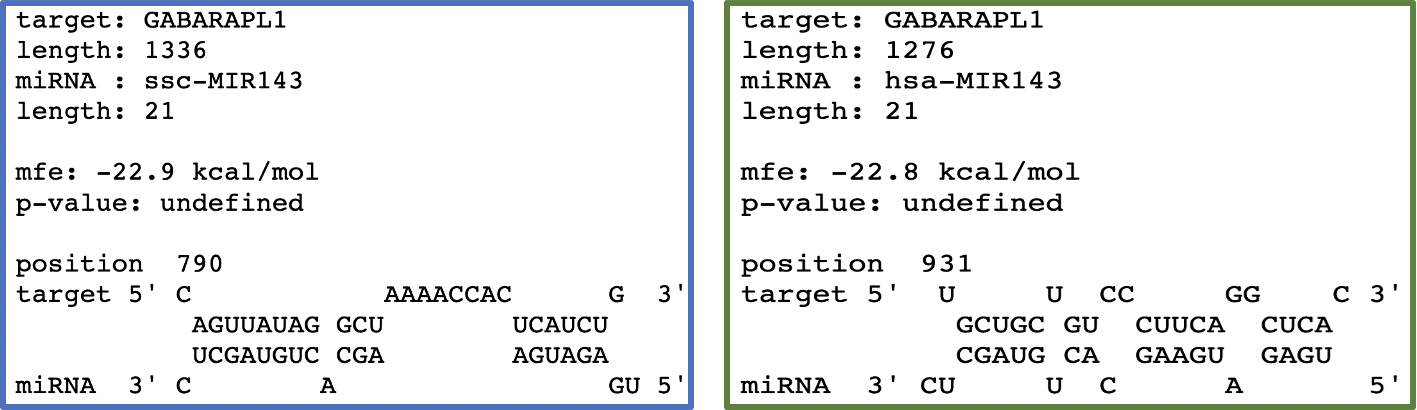
**

**F. HRAS**


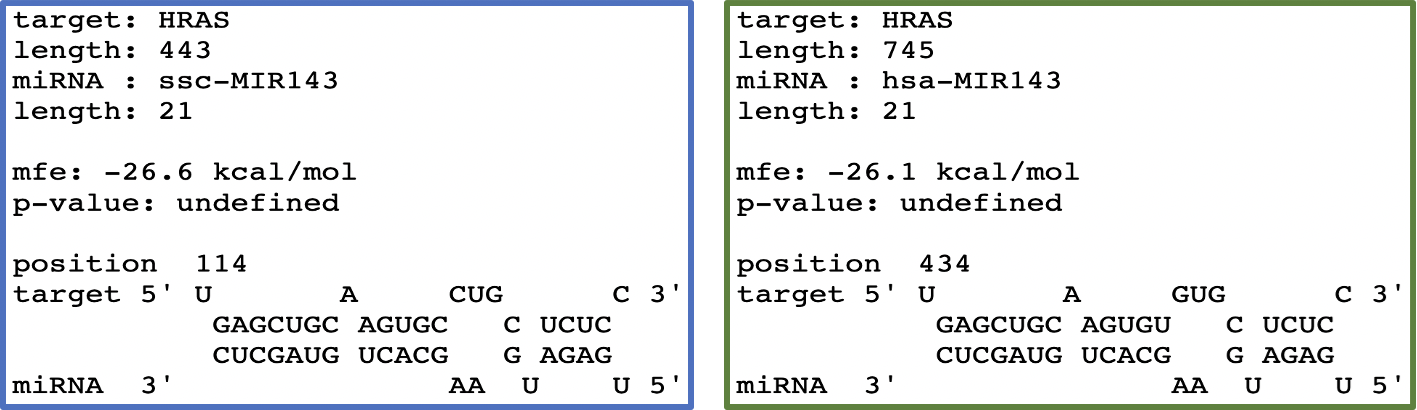


**G. IGF1R**


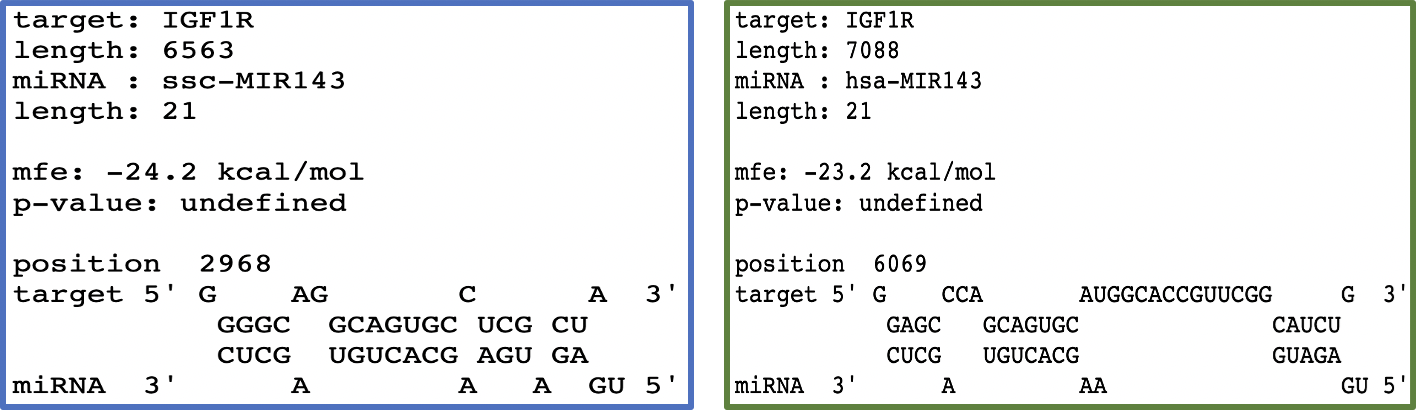


**H. KRAS**


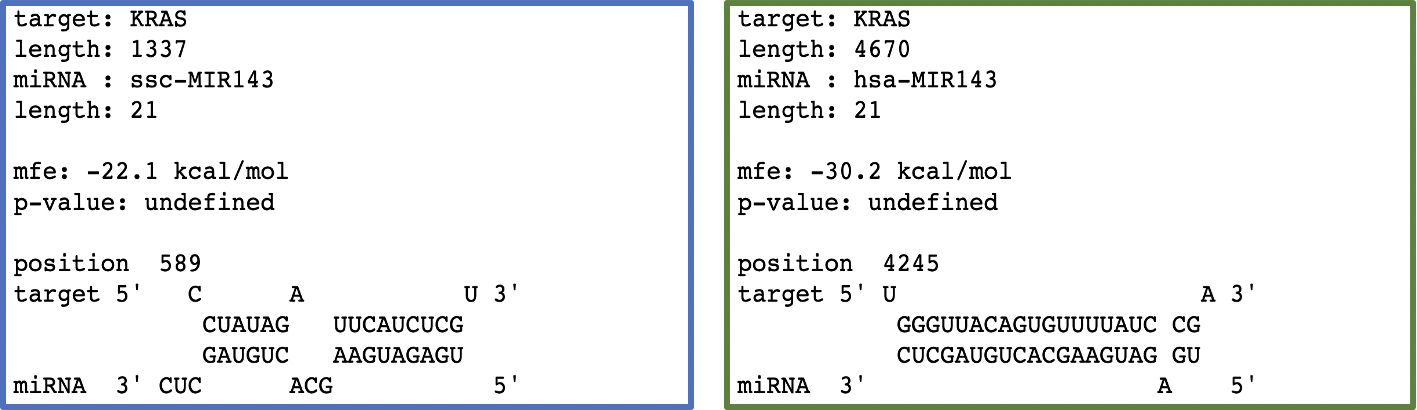


**I. MAPK7**


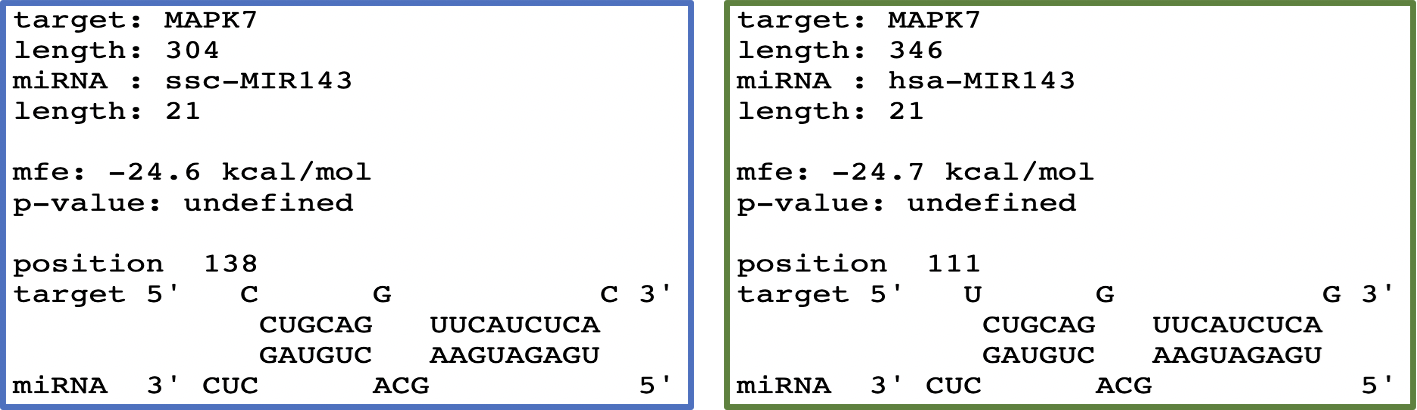


**J. MDM2**


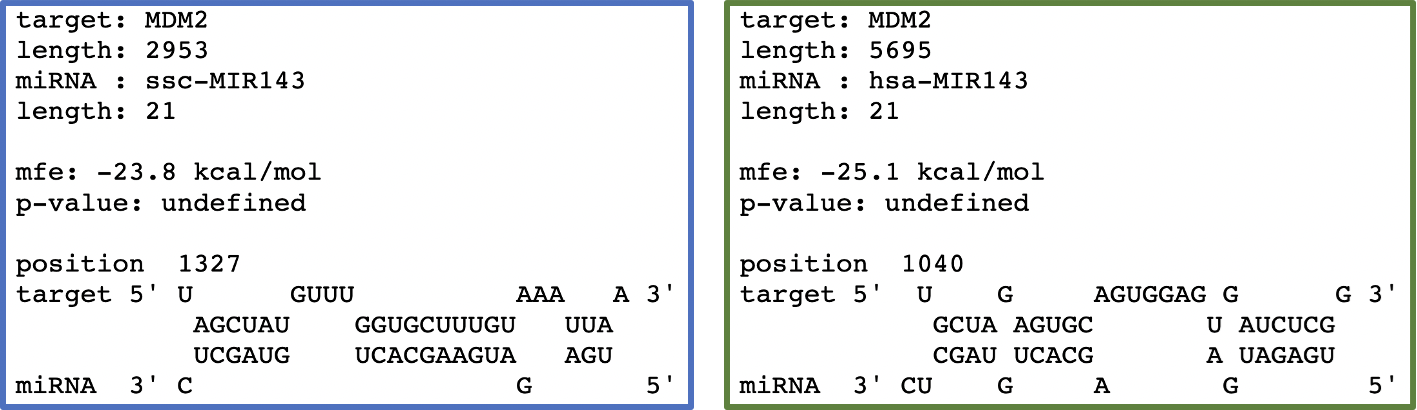


**K. MMP2**


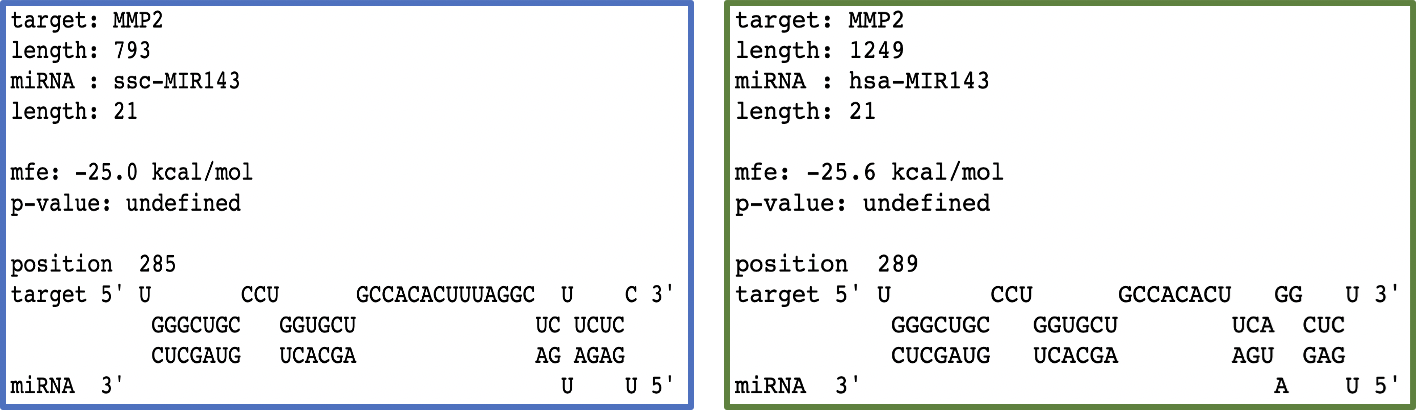


**L. MMP9**


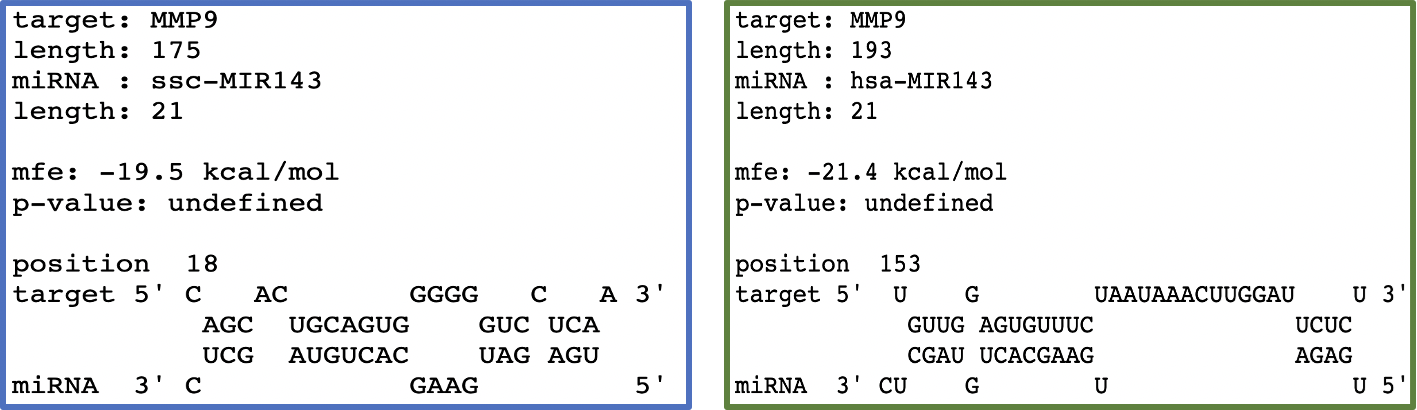


**M. MMP14**


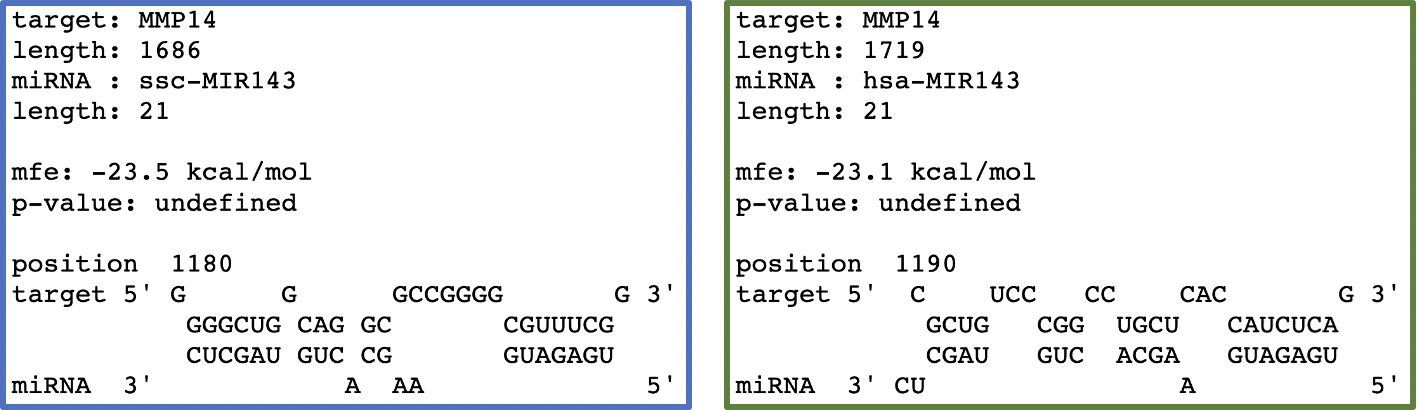


**N. NFKB1**


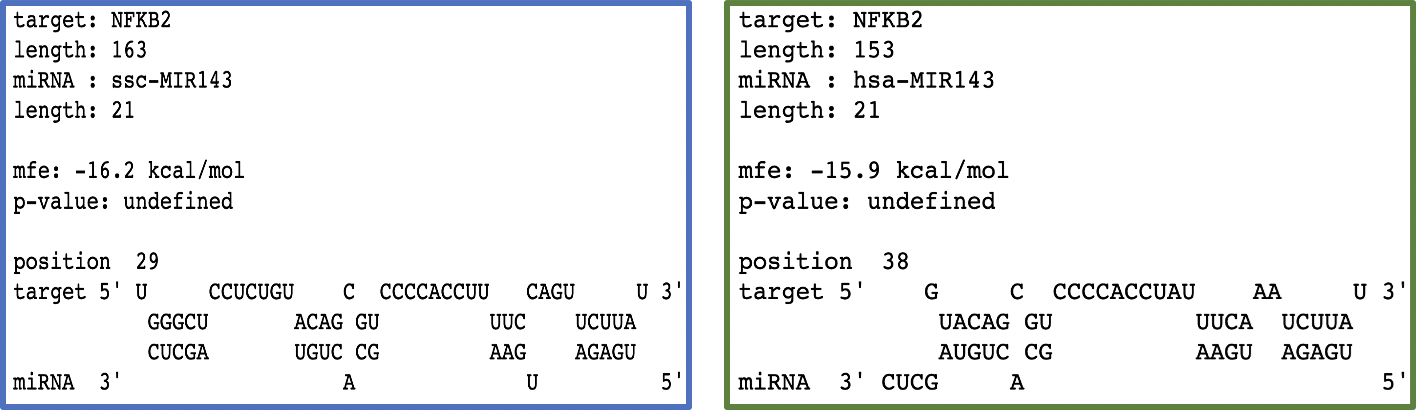


**O. PTGS2**


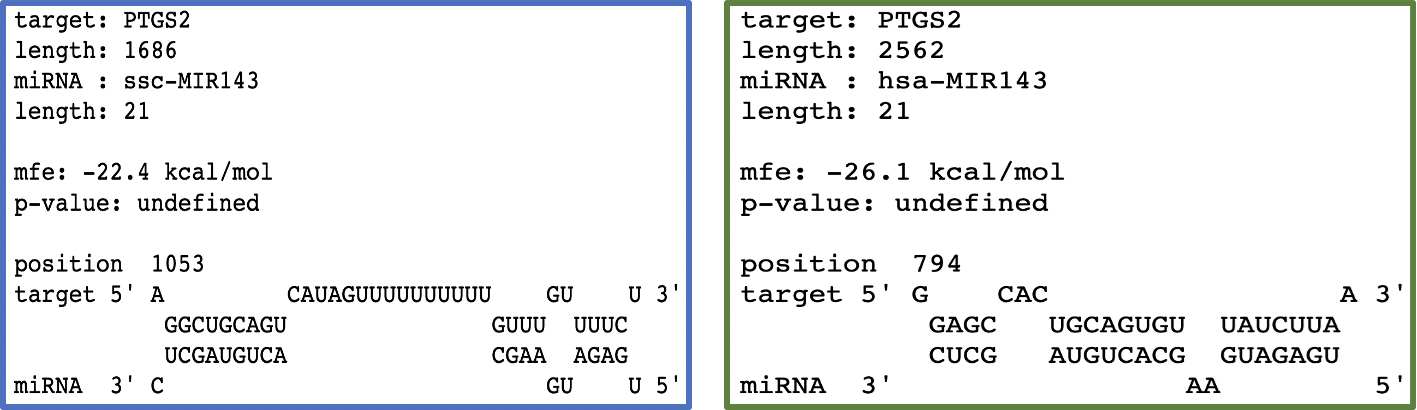


**P. *TNF***


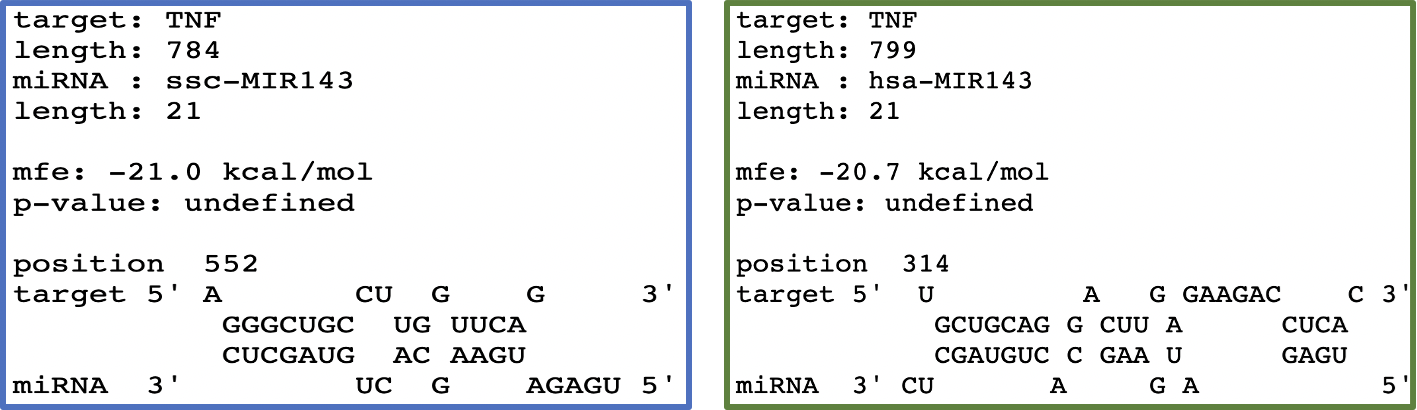


**Supplementary Figure 2.** There were16 genes predicted to be the candidate target of pig miR-143-3p (MIR143). These genes participated in the pathways related follicular development and maturation, such as progesterone-mediated oocyte maturation (KEGG: 04914; AKT1, AKT2, BRAF, IGF1R and KRAS), GnRH signaling pathway (KEGG: 04912; HRAS, KRAS, MAPK7, MMP14 and MMP2) and Estrogen signaling pathway (KEGG: 04915; AKT1, AKT2, BCL2, HRAS, KRAS, MMP2 and MMP9). Consequently, this inferred a direction to further investigate the function of MIR143-target gene signaling pathways in porcine follicular cells and follicles.
