# Supplementary material for: MIR143 Inhibits Steroidogenesis and Induces Apoptosis Repressed by H3K27me3 in Granulosa Cells
Source: Front Cell Dev Biol. 2020 Oct 19;8:565261. doi: 10.3389/fcell.2020.565261 (PMC7604341; doi:10.3389/fcell.2020.565261)
Supplement: Supplementary file 2 [file Table_2.DOCX]

**Supplementary Table 2.** The 46 verified targets of has-miR-143-3p

| miRNA | Target Genes |
| --- | --- |
| Has-miR-143-3p | *DDX6*, *SERPINE*, *ITGB4*, *GABARAPL1*, *YP2C9*, *NFKB2*, *ITGB1*, *BAG3*, *GTGF*, *NFATC1*, *IL13RA1*, *NR2C2*, *COL3A1*, *MYO6*, *IGF1R*, *TNF*, *COL1A1*, *MMP14*, *HRAS*, *TLR2*, *CDD4*, *MMP13*, *HNF4A*, *KLF5*, *MMP9*, *HKI*, *MACC1*, *BRAF*, *SDC1*, *MMP2*, *FHIT*, *OSBP8*, *BCL2*, *RREB1*, *MDM2*, *DNTB*, *FAM83F*, *AKT2*, *PTGS2*, *LIMK1*, *AKT1*, *DNMT3A*, *FNDC3B*, *MAPK7*, *KRAS*, *FSCN1* |

The 46 verified targets of human has-miR-143-3p were found in miRTarbase miRTarbas (<http://mirtarbase.mbc.nctu.edu.tw/php/search.php>), which were used to bioinformatic prediction in RNAhybrid (version 2.2.1, https://bibiserv.cebitec.uni- bielefeld.de/rnahybrid) and KEGG pathway analysis via Cytoscape/ClueGO (3.7.2)as candidate targets of MIR143.
